# Gene Variation at Immunomodulatory and Cell Adhesion Molecules Loci Impacts Primary Sjögren's Syndrome

**DOI:** 10.3389/fmed.2022.822290

**Published:** 2022-03-18

**Authors:** Sergi Casadó-Llombart, Hoda Gheitasi, Silvia Ariño, Marta Consuegra-Fernández, Noelia Armiger-Borràs, Belchin Kostov, Manuel Ramos-Casals, Pilar Brito-Zerón, Francisco Lozano

**Affiliations:** ^1^Immunoreceptors del Sistema Innat i Adaptatiu, Institut d'Investigacions Biomèdiques August Pi i Sunyer (IDIBAPS), Barcelona, Spain; ^2^Department of Autoimmune Diseases, ICMiD, Hospital Clínic, University of Barcelona, Barcelona, Spain; ^3^Primary Care Centre Les Corts, Consorci d'Atenció Primària de Salut Barcelona Esquerra (CAPSBE), Barcelona, Spain; ^4^Primary Healthcare Transversal Research Group, Institut d'Investigacions Biomèdiques August Pi i Sunyer (IDIBAPS), Barcelona, Spain; ^5^Department of Statistics and Operations Research, Universitat Politècnica de Catalunya (UPC), Barcelona, Spain; ^6^Research and Innovation Group in Autoimmune Diseases, RGAD-Sanitas Digital Hospital, Barcelona, Spain; ^7^Systemic Autoimmune Diseases Unit, Internal Medicine, Millenium Clinic, Sanitas, Barcelona, Spain; ^8^Servei d'Immunologia, Centre de Diagnòstic Biomèdic, Hospital Clínic de Barcelona, Barcelona, Spain; ^9^Departament de Biomedicina, Facultat de Medicina, Universitat de Barcelona, Barcelona, Spain

**Keywords:** Sjögren's syndrome, *CD5*, *CD6*, CD166/ALCAM, polymorphism, SNP

## Abstract

Primary Sjögren's syndrome (pSS) is an autoimmune disease triggered by a combination of environmental and host genetic factors, which results in the focal lymphocytic infiltration of exocrine glands causing eye and mouth dryness. Glandular infiltrates include T and B cell subsets positive for CD5 and/or CD6, two surface scavenger receptors involved in the fine-tuning of intracellular signals mediated by the antigen-specific receptor complex of T (TCR) and B (BCR) cells. Moreover, the epithelial cells of inflamed glands overexpress CD166/ALCAM, a CD6 ligand involved in homo and heterotypic cell adhesion interactions. All this, together with the reported association of functionally relevant single nucleotide polymorphisms (SNPs) of *CD5, CD6*, and *CD166/ALCAM* with the risk or prognosis of some immune-mediated inflammatory disorders, led us to investigate similar associations in a local cohort of patients with pSS. The logistic regression analyses of individual SNPs showed the association of *CD5* rs2241002^T^ with anti-Ro/La positivity, *CD6* rs17824933^C^ with neutropenia, and *CD6* rs11230563^T^ with increased leukopenia and neutropenia but decreased peripheral nervous system EULAR Sjögren's syndrome disease activity index (ESSDAI). Further analyses showed the association of haplotypes from *CD5* (rs2241002^T^-rs2229177^C^) with anemia and thrombocytopenia, *CD6* (rs17824933^G^-rs11230563^C^-rs12360861^G^) with cutaneous ESSDAI, and *CD166/ALCAM* (rs6437585^C^-rs579565^A^-rs1044243^C^ and rs6437585^C^-rs579565^G^-rs1044243^T^) with disease susceptibility and several analytical parameters (anti-nuclear antibodies, neurological ESSDAI, and hematologic cytopenias). These results support the relevance of gene variation at loci coding for cell surface receptors involved in the modulation of T and B lymphocyte activation (*CD5, CD6*) and epithelial-immune cell adhesion (*CD166/ALCAM*) in modulating the clinical and analytical outcomes in patients with pSS.

## Introduction

Primary Sjögren's syndrome (pSS) is a chronic, systemic rheumatic disease characterized by the lymphoplasmacytic infiltration of exocrine glands—mainly salivary and lacrimal glands—resulting in sicca syndrome and systemic manifestations (1). It is a common disorder (prevalence of 0.5–1% in the general population) with a female/male ratio of approximately 9:1 (2, 3). pSS is considered as a complex and multifactorial process whose pathogenesis involves environmental factors, such as viral infections, combined with sex hormonal, genetic and epigenetic factors, causing epithelial cell barrier disruption followed by an abnormal immune cell-mediated inflammatory response (4, 5).

Periductal immune cell infiltrates in the affected glands of patients with pSS include CD5- and/or CD6-positive T and B cells (6–9). CD5 and CD6 are two highly homologous lymphocyte surface receptors of the scavenger receptor cysteine-rich superfamily (SRCR-SF) (10). They are expressed on all T cells and a subset of B cells (B1a) involved in the production of polyreactive natural antibodies, and they are abnormally expanded in the peripheral blood of patients undergoing autoimmune disorders, such as pSS and systemic lupus erythematosus (SLE) (6, 10, 11). Both receptors are signal-transducing molecules that modulate intracellular activation and differentiation signals from the antigen-specific receptor complex of T (TCR) and B (BCR) cells to which CD5 and CD6 physically associate (12–14). In addition, CD5 and CD6 act as pattern recognition receptors (PRRs) by recognizing microbial-associated molecular patterns (MAMPs) from the bacterial, fungal, viral, and parasitic origin (15–17). Particularly, CD5 has been shown to interact with fungal β-glucans (18), hepatitis C virus (19), and tegumental structures of *Echinococcus granulosus* (20), while CD6 interacts with lipopolysaccharide, lipoteichoic acid, and peptidoglycan from Gram-negative and -positive bacteria (21), gp120 from human immunodeficiency virus 1 (22), and the tegumental components of *E. granulosus* (20).

A central phenomenon in the immunopathogenesis of pSS is the aberrant epithelial cell activation status (pSS has been described as an autoimmune epithelitis) (23, 24). This results in the increased expression of human leukocyte antigen (HLA)-DR, costimulatory, and adhesion molecules. Among the latter, overexpression of the well-known CD6 ligand CD166/ALCAM has been reported in pSS epithelial lesions (8, 9, 25). CD166/ALCAM (for activated leukocyte cell adhesion molecule) is an adhesion molecule of the immunoglobulin superfamily with a broad tissue distribution, such as epithelia, endothelia, neurons, myeloid progenitors, hematopoietic stem cells, mesenchymal stem cells, bone marrow stromal cells, and cancer cells (26). Interestingly, CD166/ALCAM establishes not only homophilic (ALCAM-ALCAM) but also higher affinity heterophilic (ALCAM-CD6) interactions with the CD6 lymphocyte receptor, which facilitate cell interactions of T or B1a lymphocytes with epithelial and endothelial cells (26–28).

Studies aimed at the genetic basis of pSS show the associations of both human leukocyte antigen (HLA) and non-HLA genes with pSS susceptibility. The HLA-DR and HLA-DQ alleles have shown the strongest associations across different ethnicities (29, 30). The long, though still incomplete, list of non-HLA genetic polymorphisms contributed by genome-wide (GWAS) and gene-driven association studies includes interferon regulatory factor 5 (IRF5), signal transducer and activator of transcription 4 (STAT4), B lymphocyte kinase (BLK), tumor necrosis factor-α (TNF-α), interleukin (IL)-4, IL-10, IL-12A, C-X-C chemokine receptor type 5 (CXCR5), surfactant protein-D (SP-D), and Mannan-binding lectin (MBL) (30–36).

Single nucleotide polymorphisms (SNPs) at the *CD5, CD6*, and *CD166/ALCAM* gene loci have been associated with different immune-mediated inflammatory diseases (IMID) (37). Specifically, *CD5* variation has been associated with rheumatoid arthritis (RA) susceptibility (38) and the development of lupus nephritis (39). *CD6* and *CD166/ALCAM* SNPs have been identified and validated as risk factors for the development and progression of multiple sclerosis (MS) (40–42), psoriasis severity (43), Behçet's disease risk (44), and inflammatory bowel disease (IBD) risk (45, 46).

Given the expression of CD5, CD6, and CD166/ALCAM in pSS inflamed tissue and the association of their SNPs with other IMIDs, we hypothesize that variation at *CD5, CD6*, and *CD166/ALCAM* loci may impact the pathology of pSS. The results of the present candidate gene-driven association analysis show that *CD5, CD6*, and *CD166/ALCAM* genetic polymorphisms are associated with the clinical and analytical parameters of the disease in a local cohort of pSS patients.

## Materials and Methods

### Subjects

Consecutive patients with pSS (*n* = 212) attending to the Hospital Clínic de Barcelona, Barcelona, Spain were included in the study (Table 1). Patients fulfilled the 2002/2016 criteria approved by the American-European Consensus Group (47). Exclusion criteria for considering SS as a primary disease were chronic HCV/HIV infection, previous lymphoproliferative processes, and associated systemic autoimmune diseases. Diagnostic tests for SS (ocular tests, parotid scintigraphy, and salivary gland biopsy) were performed according to the European Community Study Group recommendations (48).

**Table 1:** General characteristics of the primary Sjögren's syndrome (pSS) cohort.

**Variables**	***n* (%)**
Gender (Female)	202 (95.3)
Ethnicity (Caucasian)	201 (94.8)
Age at diagnosis	54 (14.4)
Dry mouth	212 (100)
Dry eyes	205 (96.7)
Schirmer test (abnormal)	185/194 (95.4)
Salivary scintigrahy (abnormal)	163/180 (90.6)
Minor salivary gland biopsy (positive)	103/113 (91.2)
Antinuclear antibodies (positive)	181/211 (85.8)
Rheumatoid factor (positive)	98/208 (47.1)
Anti-Ro/La antibodies (positive)	151 (71.2)
Anti-Ro	143 (67.5)
Anti-La	103/211 (48.8)
Monoclonal gammopathy	25/142 (17.6)
Low C3 levels (<0.82 g/L)	19/210 (9)
Low C4 levels (<0.11 g/L)	13/207 (6.3)
Cryoglobulins	17/201 (8.5)
Cytopenias	109/211 (51.7)
Anemia (Hb <110 g/L)	43/211 (20.4)
Leukopenia (<4,000/mm^3^)	57/211 (27)
Thrombocytopenia (<150,000/mm^3^)	23/211 (10.9)
Neutropenia (<1,500/mm^3^)	53/211 (25.1)
Lymphopenia (<1,000/mm^3^)	21/211 (10)
ESSDAI domains (activity)	
Constitutional	28 (13.2)
Lymphadenopathy	27 (12.7)
Glandular	60 (28.3)
Articular	93 (43.9)
Cutaneous	37 (17.5)
Pulmonary	41 (19.3)
Renal	5 (2.4)
Muscular	1 (0.5)
Peripheral nervous system	23 (10.8)
Central nervous system	8 (3.8)
Hematological	159 (75)
Biological	141 (66.5)
Total ESSDAI (baseline)	7.4 (6.8)
Total ESSDAI (cumulative)	10.2 (8.5)

Unrelated volunteers (*n* = 305) from the Banc de Sang i Teixits (BST) from Generalitat de Catalunya were included as controls (143 women and 162 men).

The study was approved by the local Hospital Ethics Committee, and written informed consent was obtained from all participants before inclusion and blood extraction.

### Definition of Variables

Disease diagnosis was defined as the time when the attending physician confirmed the fulfillment of the 2002/2016 criteria (47). The main disease features were retrospectively collected and analyzed. The following clinical variables were selected for harmonization and further refinement: age, gender, ethnicity, country of residence, fulfillment of the 2002/2016 criteria items, antinuclear antibodies (ANA), rheumatoid factor (RF), C3 and C4 levels, and cryoglobulins. The epidemiological variables included in this study were age at diagnosis, gender, and ethnicity according to the Food and Drug Administration (FDA) definitions (49). Systemic involvement at diagnosis was retrospectively classified and scored according to the EULAR Sjögren's syndrome disease activity index (ESSDAI) (50), which evaluates 12 domains or organ systems, and the ClinESSDAI (51), which evaluates the same domains but excluding the last (biological) domain. Each domain is divided into 3–4 levels according to the degree of activity and scored as 0 (no activity), 1 (low activity), 2 (moderate activity), or 3 (high activity) (52). Disease activity states (DAS) were calculated as: no activity (global score = 0), low activity (global score 1–4), moderate activity (global score 5–13), and high activity (global score ≥14) (53).

Additionally, cumulative systemic involvement was classified and scored according to the ESSDAI. Cumulative systemic involvement was defined as the systemic activity present since the diagnosis of pSS to the last medical visit.

### Genotyping

DNA was purified from ethylenediaminetetraacetic acid (EDTA)-treated peripheral blood using the QIAamp DNA Blood Mini Kit (Qiagen, Venio, The Netherlands) and subjected to real-time (RT)-PCR with the following TaqMan probes: *CD5* rs2241002 (assay number: C__25472293_20), *CD5* rs2229177 (assay number: C___3237272_10), *CD6* rs17824933 (assay number: C__33967506_10), *CD6* rs11230563 (assay number: C__31727142_10), *CD6* rs12360861 (assay number: C__25922320_10), and *CD166/ALCAM* rs6437585 (assay number: C__29281365_20), all from ThermoFisher Scientific (Barcelona, Spain). Primers for PCR amplification and further sequence-base typing (PCR-SBT) of *CD166/ALCAM* rs579565 and rs1044243, which lie 2 bp apart from each other, were also from ThermoFisher Scientific (Hs00666884_CE assay). SNP genotyping and clinical data are available at a public repository (54).

### Statistical Analyses

Statistical analyses were performed with R 3.6.0 (R Foundation for Statistical Computing, Vienna, Austria). Genotypic statistical associations among the SNPs and susceptibility or disease outcomes were tested by generalized linear models using the R package “SNPstats.” For each analysis, 4 models were generated (codominant, dominant, recessive, and log-additive), and the model with the lowest Akaike information criterion (AIC) was chosen. The *p* values were corrected for false discovery rate (FDR, *q* values). Haplotypic analyses were performed with generalized linear models by means of the R package “haplo.stats.”

## Results

A total of 212 patients with pSS with a mean age of 54 years at diagnosis were included in the study, most of them were women (95.3%) and presented dry mouth (100%) and dry eyes (96.7%). The association of individual SNPs with susceptibility and the clinical parameters of pSS was first investigated (Supplementary Table 1). Sex is a major risk factor in pSS, so statistical models for subphenotypical analyses were generated with or without including sex as a covariant, and their goodness of fit compared with the AIC. The results presented here do not include sex as a covariant, as these models had lower AIC. Susceptibility analyses were performed only with female patient cases and controls. No significant association was found between any individual *CD5, CD6*, and *CD166/ALCAM* SNPs and pSS susceptibility, although the *CD166/ALCAM* rs579565^A^ allele showed a trend for statistical association in women (*q* = 0.064) (Table 2).

**Table 2:** Logistic regression analyses of *CD166/ALCAM* SNP association with pSS susceptibility.

**Gene**	**SNP**	**Model**	**Genotype**	**Controls (%)**	**pSS cases (%)**	**OR (95% CI)**	***q* value**
*CD166/* *ALCAM*	rs579565	Recessive	G/G-G/A A/A	139 (97.9) 3 (2.1)	169 (91.4) 16 (8.6)	4.39 (1.25, 15.36)	0.064

Regarding association with pSS clinical parameters, the *CD5* rs2241002^C^ allele was found associated with a higher frequency of anti-Ro/La antibody positivity (Table 3). The *CD6* rs17824933^G^ allele was associated with decreased risk of neutropenia (Table 3), and the *CD6* rs11230563^T^ allele with increased leukopenia and neutropenia, but decreased ESSDAI peripheral nervous system (PNS) activity (Table 3).

**Table 3:** Logistic regression analyses of *CD5* and *CD6* SNPs association with anti-Ro/La antibodies, neutropenia, leukopenia, and peripheral nervous system (PNS) EULAR Sjögren's syndrome disease activity index (ESSDAI) activity.

**Gene**	**SNP**	**Model**	**Genotype**	**No anti-Ro/La (%)**	**Anti-Ro/La (%)**	**OR (95% CI)**	***q* value**
*CD5*	rs2241002	Recessive	C/C-C/T	55 (90.2)	149 (98.7)		0.046
			T/T	6 (9.8)	2 (1.3)	0.12 (0.02, 0.63)	
				**No neutropenia (%)**	**Neutropenia (%)**		
*CD6*	rs17824933	Dominant	C/C C/G-G/G	75 (50.3) 74 (49.7)	33 (73.3) 12 (26.7)	0.37 (0.18, 0.77)	0.022
				**No leukopenia (%)**	**Leukopenia (%)**		
	rs11230563	Recessive	C/C-C/T T/T	121 (85.8) 20 (14.2)	34 (65.4) 18 (34.6)	3.20 (1.53, 6.73)	0.019
				**No neutropenia (%)**	**Neutropenia (%)**		
		Recessive	C/C-C/T T/T	127 (85.8) 21 (14.2)	28 (62.2) 17 (37.8)	3.67 (1.72, 7.84)	0.008
				**No PNS ESSDAI activity (%)**	**PNS ESSDAI activity (%)**		
		Dominant	C/C C/T-T/T	50 (28.7) 124 (71.3)	12 (60.0) 8 (40.0)	0.27 (0.10, 0.70)	0.041

Haplotypic analyses showed the association of *CD5* rs2241002^T^-rs2229177^C^ haplotype with an increased risk of anemia and thrombocytopenia (Table 4). The *CD6* rs17824933^G^-rs11230563^C^-rs12360861^G^ haplotype was associated with an increased risk of ESSDAI cutaneous activity (Table 5). The *CD166/ALCAM* rs6437585^C^-rs579565^G^-rs1044243^T^ haplotype was associated with increased ANA positivity, ESSDAI PNS activity, and hematologic cytopenias, such as anemia and lymphopenia (Table 6).

**Table 4:** Logistic regression analysis of CD5 haplotype association to anemia and thrombocytopenia.

**Haplotype**			**Anemia**			
**rs2241002**	**rs2229177**	**% in cohort**	**% No**	**% Yes**	***p* value**	**OR (95% CI)**
C	C	41.0	41.8	39.9		
C	T	39.1	38.9	38.0	0.941	1.02 (0.58, 1.78)
T	T	15.2	16.5	12.0	0.399	0.69 (0.29, 1.62)
**T**	**C**	4.7	2.8	10.0	**0.032**	4.48 (1.14, 17.60)
		**% in cohort**	**Thrombocytopenia**			
			**% No**	**% Yes**		
C	C	41.0	39.7	35.9		
C	T	39.1	41.4	35.9	0.905	1.07 (0.46, 2.50)
T	T	15.2	15.1	14.1	0.872	0.93 (0.27, 3.19)
**T**	**C**	4.7	3.8	14.1	**0.036**	5.83 (1.12, 30.29)

*Significant haplotypes are shown in bold*.

**Table 5:** Logistic regression analysis of *CD6* haplotype association to cutaneous affectation. Only the 4 most common haplotypes are shown.

**Haplotype**				**Cutaneous ESSDAI activity**			
**rs17824933**	**rs11230563**	**rs12360861**	**% in cohort**	**% No**	**% Yes**	***p* value**	**OR (95% CI)**
C	C	G	32.6	35.2	20.1		
**G**	**C**	**G**	23.6	22.1	31.4	**0.012**	2.85 (1.26, 6.43)
C	T	A	22.4	22.3	20.8	0.141	1.81 (0.82, 4.00)
C	T	G	20.6	20.0	25.8	0.055	2.26 (0.98, 5.12)

*Significant haplotypes are shown in bold*.

**Table 6:** Logistic regression analysis of *CD166/ALCAM* haplotype association with anti-nuclear antibodies (ANA), cytopenia, anemia, lymphopenia, peripheral nervous system (PNS) ESSDAI activity, and pSS susceptibility.

**Haplotype**				**ANA positivity**		***p* value**	**OR (95% CI)**
**rs6437585**	**rs579565**	**rs1044243**	**% in cohort**	**% Negative**	**% Positive**		
C	G	C	55.1	58.4	54.3		
C	A	C	27.3	30.5	27.2	0.685	0.87 (0.46, 1.66)
**C**	**G**	**T**	13.0	3.7	14.3	**0.045**	4.64 (1.04, 20.81)
T	G	C	3.0	4.5	3.0	0.494	0.57 (0.11, 2.90)
				**Cytopenia**			
			**% in cohort**	**% No**	**% Yes**		
C	G	C	55.4	58.0	53.1		
C	A	C	27.1	27.8	26.3	0.975	0.97 (0.61, 1.54)
**C**	**G**	**T**	12.8	9.0	16.5	**0.027**	2.14 (1.08, 4.21)
T	G	C	3.0	3.5	3.0	0.657	0.71 (0.16, 3.15)
				**Anemia**			
			**% in cohort**	**% No**	**% Yes**		
C	G	C	55.4	57.1	49.0		
C	A	C	27.1	26.7	27.6	0.632	1.15 (0.65, 2.06)
**C**	**G**	**T**	12.8	11.0	20.7	**0.030**	2.25 (1.08, 4.66)
T	G	C	3.0	3.6	0.0	–	–
				**Lymphopenia**			
			**% in cohort**	**% No**	**% Yes**		
C	G	C	55.4	56.1	56.3		
C	A	C	27.1	27.4	18.8	0.907	0.95 (0.44, 2.08)
**C**	**G**	**T**	12.8	11.3	18.8	**0.030**	2.64 (1.10, 6.35)
T	G	C	3.0	3.2	0.0	–	–
				**PNS ESSDAI activity**			
			**% in cohort**	**% No**	**% Yes**		
C	G	C	55.1	55.6	52.5		
C	A	C	27.3	28.1	20.0	0.404	0.70 (0.30, 1.62)
**C**	**G**	**T**	13.0	11.5	25.0	**0.036**	2.56 (1.06, 6.15)
T	G	C	3.0	3.1	0.0	–	–
				**pSS susceptibility**			
			**% in pool**	**% controls**	**% cases**		
C	G	C	58.5	63.3	54.8		
**C**	**A**	**C**	24.4	20.6	27.3	**0.044**	1.51 (1.01, 2.24)
**C**	**G**	**T**	11.4	8.8	13.3	**0.046**	1.72 (1.01, 2.95)
T	G	C	4.2	5.8	2.7	0.274	0.62 (0.26, 1.47)

*Only the 4 most common haplotypes are shown. Significant haplotypes are shown in bold*.

Case-control analyses to assess the influence of *CD5, CD6*, and *CD166/ALCAM* haplotypes on pSS risk were also performed. To account for the gender skew in pSS, only female cases and controls were included in this haplotypic analysis. The results showed that the only associations with pSS susceptibility were with the *CD166/ALCAM* rs643785^C^-rs579565^A^-rs1044243^C^ (CAC) and rs643785^C^-rs579565^G^-rs1044243^T^ (CGT) haplotypes (Table 6), which were over-represented in the case cohort, indicating the association of rs579565^A^ and rs1044243^T^ alleles with pSS susceptibility.

## Discussion

The pathophysiology of pSS is complex and multifactorial. How the innate and adaptive immune responses are dysregulated through both cellular- and humoral-mediated processes (30) is still poorly understood. Identifying genetic factors associated with pSS may help in the better comprehension of pathogenic mechanisms leading to the overall pSS phenotype and clinically heterogeneous subsets of patients (55). By using a candidate gene-driven strategy, the present work shows evidence on the impact of *CD5, CD6*, and *CD166/ALCAM* gene variants in the susceptibility and clinical expression of pSS, thus supporting their involvement in pSS pathophysiology.

*CD5, CD6*, and *CD166/ALCAM* variation study in pSS responds to: first, the three genes encode functionally relevant and related cell surface receptors. CD5 and CD6 are highly homologous lymphocyte receptors of the ancient and highly conserved SRCR-SF and are encoded by contiguous genes likely resulting from a duplication event (56, 57). Both CD5 and CD6 are expressed by all T cell types and the B1a cell subset, with the lower levels of expression in other cell types (e.g., macrophages, dendritic cells, or natural killer cells) (10, 13), all found in pSS periductal immune cell infiltrates (6–9). From the functional point of view, CD5 and CD6 are considered relevant signaling immune receptors at the interphase of the innate and adaptive immune responses as a result from their involvement in (*i*) the recognition and sensing of bacterial, viral, and/or parasitic MAMPs (17) and (*ii*) the fine-tuning of lymphocyte activation signals delivered by clonotypic T and B antigen-specific receptors, which they are physically associated to (58–60). While the nature of the endogenous CD5 ligand is yet uncertain, one of the most-well studied CD6 ligands is CD166/ALCAM, a cell adhesion molecule overexpressed in pSS salivary gland epithelial cells (8, 9, 25), but also RA synovium (61), MS blood–brain barrier endothelium (62), and lupus nephritis kidneys (63), thus contributing to T and B cell migration and infiltration at inflamed tissues in autoimmune processes.

Second, several *CD5, CD6*, and/or *CD166/ALCAM* gene variants have been associated with different IMIDs, such as RA (38), lupus nephritis (39), MS (40–42), psoriasis (43), Behçet's disease (44), and IBD (45, 46) (Supplementary Table 2). The *CD5, CD6*, and *CD166/ALCAM* SNPs included in the present study were selected not only for being informative in the above-mentioned IMIDs but also for their putative functional relevance. Regarding CD5, the rs2241002 (C > T) and rs2229177 (C > T) SNPs result in amino acid substitutions at the extracellular SRCR2 domain (Pro224>Leu) and just next to a cytoplasmic ITAM-like motif (Ala471>Val), respectively (39, 64). Functional studies show that homozygous carriers for the ancestral rs2241002^C^-rs2229177^C^ haplotype (Pro224-Ala471) present increased T-cell proliferation and cytokine release and a bias toward a Th2 profile, compared with the homozygous carriers of more recently derived rs2241002^C^-rs2229177^T^ haplotype (Pro224-Val471) (39). Regarding CD6, the rs11230563 (C>T) and rs12360861 (G>A) SNPs result in amino acid substitutions at the extracellular SRCR2 (Arg225>Trp) and SRCR3 (Ala271>Thr) domains, respectively, and the intron 1 rs17824933 (C>G) SNP results in the skipping of exon 5 and expression of a CD6 isoform lacking the SRCR3 domain (CD6Δd3), in which the CD166/ALCAM-binding site locates (65). Functional studies show that the *CD6* rs11230563^C^-rs2074225^C^ haplotype (Arg225-Ala257) results in higher CD6 surface expression on CD4^+^ and CD8^+^ naïve T cells and NKT cells (41). The carriage of *CD6* rs17824933^G^ allele results in an increased CD6Δd3/full-length CD6 ratio driving to lower CD4^+^ T cell activation responses (66). Regarding *CD166/ALCAM*, the rs6437585 (C > T) SNP maps at the 5'-untranslated region (UTR) and is known to influence the transcriptional activity of *CD166/ALCAM* (42, 67), while the rs579565 (G > A) and rs1044243 (C > T) SNPs result in synonymous (Leu300>Leu) and non-synonymous (Thr301>Met) changes at the extracellular C1-like domain (42) with still unknown functional consequences.

Individual SNP and haplotypic analyses showed the association of *CD5, CD6*, and *CD166/ALCAM* SNPs with different pSS clinical parameters. Thus, the *CD5* rs2241002^C^ allele and the minor *CD5* rs2241002^T^-rs2229177^C^ haplotype, previously associated with a more aggressive form of SLE (lupus nephritis) (39), showed association with anti-Ro/anti-La antibody positivity, and with anemia and thrombocytopenia, respectively. This could be interpreted as a result of hyperactive autoantibody-producing B cells (most likely CD5^+^ B1a cells) in pSS carriers of such *CD5* variants.

The individual *CD6* rs11230563^C^ allele was associated with the higher risk of PNS ESSDAI activity, and the *CD6* rs17824933^G^-rs11230563^C^-rs12360861^G^ haplotype with cutaneous ESSDAI activity. This is reminiscent of the increased MS risk and psoriasis severity previously reported for rs11230563^C^ allele (40, 43, 68–70). It is noteworthy that both rs17824933^G^ and rs11230563^C^ alleles were associated with the reduced risk of neutropenia. Since both alleles impact the extracellular region of CD6 (an increased expression of CD6Δd3 isoform and Arg225 to Trp substitution at SRCR2, respectively), it remains to be analyzed whether this relates to the reported surface CD6 (and CD166/ALCAM) expression by hematopoietic cell progenitors present in the bone marrow and in mobilized blood (71, 72).

The *CD166/ALCAM* (rs6437585^C^-rs579565^G^-rs1044243^T^) haplotype was found associated with the increased incidences of ANA positivity, neurological affectation, and hematologic cytopenias. These results further support the damaging role of *CD6* rs17824933^G^ and rs11230563^C^ alleles and of *CD166/ALCAM* rs1044243^T^ allele by worsening some analytical and clinical parameters of pSS. Interestingly, haplotypic analyses showed the association of *CD166/ALCAM* rs6437585^C^-rs579565^A^-rs1044243^C^ and rs6437585^C^-rs579565^G^-rs1044243^T^ haplotypes with increased pSS susceptibility. This supports a role for minor rs579565^A^ and rs1044243^T^ alleles in pSS susceptibility, which is reminiscent of the earlier age of MS diagnosis reported for the rs579565^A^ allele (42).

The association of *CD5, CD6*, and *CD166/ALCAM* SNPs with pSS phenotype highlights the relevance of genetic variation at loci related with immune activation in pSS pathophysiology. In addition, this is illustrated by the previously reported association of HLA-DR and HLA-DQ, *IRF5, STAT4, BLK, TNF, IL4RA, IL10, IL12A, CXCR5, TNFAIP3, MTHFR, CD28, CTLA4, IKZF1, HIF1A, AKNA, SFTPD*, and *MBL2* loci with pSS (30–36, 73, 74) (Supplementary Table 3). Interestingly, CD5 and CD6 interact with microorganisms, such as SP-D and mannose-binding lectin (encoded by *SFTPD* and *MBL*, respectively). This brings out the relevance of microbial/pathogen recognition in pSS.

We are aware of some limitations in the present study regarding: first, the limited number of pSS cases and controls in this single-center study. Second, only a single patient cohort was available for the analysis in spite of our efforts to access validation cohorts with the necessary subphenotypical data for replicates. Therefore, validation in an independent cohort is pending for significant confirmation of the role of *CD5, CD6*, and *CD166*/*ALCAM* gene variants in pSS.

In summary, we identified the *CD166/ALCAM* rs579565 and rs1044243 SNPs as pSS risk markers, and the *CD5* rs2241002, *CD6* rs17824933 and rs11230563 and *CD166/ALCAM* rs1044243 SNPs as disease modifiers markers. Further studies in independent cohorts will be required to validate these results. Nevertheless, our observations are the first to support a role for *CD5, CD6*, and *CD166/ALCAM* variation in pSS, and they highlight the shared immunogenetic basis of different IMIDs (75). These results, along with the identification of other genetic factors involved in pSS etiopathogenesis, may also help to classify patients and allow better identification, management, and treatment of the disease.

## Data Availability Statement

The datasets presented in this study can be found in online repositories. The names of the repository/repositories and accession number(s) can be found below: https://github.com/SergiCLl/CD5-CD6-ALCAM-pSS, CD5-CD6-ALCAM-pSS.

## Ethics Statement

The studies involving human participants were reviewed and approved by Comitè d'Ètica de la Investigació amb medicaments (CEIm) Hospital Clínic de Barcelona. The patients/participants provided their written informed consent to participate in this study.

## Author Contributions

FL, MR-C, and PB-Z conceptualized the study. SC-L, HG, SA, MC-F, and NA-B contributed to genetic studies. HG, MR-C, and PB-Z contributed to sample and clinical information collection. SC-L and BK contributed to statistical analyses. SC-L, PB-Z, and FL wrote the original draft. All authors read, critically revised, and approved the final version of the manuscript.

## Funding

This work was supported by grants from the Spanish Ministerio de Economía y Competitividad (MINECO; SAF2016-80535-R) and the Ministerio de Ciencia e Innovación (MCIN/AEI/10.13039/501100011033, PID2019-106658RB-I00), co-financed by the European Development Regional Fund (A way to achieve Europe) ERDF, and Agència de Gestió d'Ajuts Universitaris i de Recerca (AGAUR; 2017/SGR/1582) from Generalitat de Catalunya. SC-L is recipient of a fellowship from the Spanish Ministerio de Educación, Cultura y Deporte (FPU15/02897) and Universitat de Barcelona (Convocatòria d'ajuts per a alumnes que estan finalitzant la tesi doctoral). Funders had no access to clinical data and did not participate in study design, data analysis, or manuscript redaction.

## Conflict of Interest

FL is a founding partner at Sepsia Therapeutics SL. The remaining authors declare that the research was conducted in the absence of any commercial or financial relationships that could be construed as a potential conflict of interest.

## Publisher's Note

All claims expressed in this article are solely those of the authors and do not necessarily represent those of their affiliated organizations, or those of the publisher, the editors and the reviewers. Any product that may be evaluated in this article, or claim that may be made by its manufacturer, is not guaranteed or endorsed by the publisher.
