# Supplementary material for: Gene Variation at Immunomodulatory and Cell Adhesion Molecules Loci Impacts Primary Sjögren's Syndrome
Source: Front Med (Lausanne). 2022 Mar 18;9:822290. doi: 10.3389/fmed.2022.822290 (PMC8971656; doi:10.3389/fmed.2022.822290)
Supplement: Supplementary file 2 [file Data_Sheet_1.docx]

Supplementary Material

# Supplementary Tables

**Supplementary Table 1.** Prevalence of pSS clinical parameters among *CD5*, *CD6* and *CD166*/*ALCAM* SNPs. See Supplementary Table 1.xslx spreadsheet.

SNP association with clinical parameters was tested by generalized linear models on the best fitting inheritance model and corrected by false discovery rate (*q* values). N (%) is shown. *Mean age at diagnosis in years (SD) is shown.

**Supplementary Table 2.** Previous studies on *CD5*, *CD6* and *CD166/ALCAM* association with other autoimmune diseases.

| **Gene** | **SNP** | **Alleles** | **Change** | **Functional/clinical relevance** |
| --- | --- | --- | --- | --- |
| *CD5* | rs2241002 | C>T | Pro224>  Leu | T allele associated with lower risk of lupus nephritis. Haplotypic combinations with rs2229177 associated to lupus nephritis [1]. |
|  | rs2229177 | C>T | Ala471>  Val | T allele associated with more signaling upon CD5 stimulation [2], stronger TCR inhibition and decreased lupus nephritis risk [1]. |
| Inter-genic | rs650258 | T>C |  | C allele associated with increased multiple sclerosis (MS) risk [3]. |
|  | rs595158 | C>A |  | Risk locus in rheumatoid arthritis [4]. |
| *CD6* | rs17824933 | C>G | Intronic | G allele associated with increased expression of CD6Δd3 [5, 6], increased MS risk in European cohorts [7–9], increased psoriasis severity [10]. |
|  | rs11230563 | C>T | Arg225>  Trp | Haplotypic combinations with rs2074225 associated with differential *CD6* expression and MS risk [3, 11]. T allele associated with decreased psoriasis severity [10] and increased Behçet’s disease risk in Han population [12]. Involvement in IBD [13, 14]. |
|  | rs2074225 | T>C | Val257>  Ala | Haplotypic combinations with rs11230563 associated to differential CD6 expression [3]. T allele associated with increased MS risk in a European cohort [3]. |
|  | rs12360861 | G>A | Ala271>  Thr | A allele associated with decreased MS risk in a European cohort [15] and increased psoriasis severity [10]. |
| *CD166/ALCAM* | rs6437585 | C>T | 5’UTR | T allele associated with higher transcriptional activity [16], earlier MS onset [15]. |
| Alleles are depicted as ancestral > derived. | | | | |

**Supplementary Table 3.** Previous studies on genetic associations with pSS.

| **Gene** | **Allele** | **Effect on pSS** |
| --- | --- | --- |
| *HLA-DQ* | DQA1*05:01 | Increased susceptibility [17, 18]. |
|  | DQB1*02:01 | Increased susceptibility [17, 18]. |
|  | DQA1*02:01 | Decreased susceptibility [17]. |
|  | DQA1*03:01 | Decreased susceptibility [17]. |
|  | DQB1*05:01 | Decreased susceptibility [17]. |
|  | rs2856674^G^ (Formerly rs115575857) | Increased susceptibility [18]. |
|  | rs3129770^G^ (Formerly rs116232857) | Increased susceptibility [18]. |
| *HLA-DR* | DRB1*03:01 | Increased susceptibility [17, 18]. |
| *IRF5* | rs3757387^C^ | Increased susceptibility [18]. |
|  | rs4728142^A^ | Increased susceptibility [18]. |
|  | rs17339836^T^ | Increased susceptibility [18]. |
|  | rs17338998^T^ | Increased susceptibility [18]. |
|  | rs10954213^G^ | Decreased susceptibility [18]. |
| *STAT4* | rs10553577^T^ | Increased susceptibility [18]. |
|  | rs13426947^A^ | Increased susceptibility [18]. |
| *IL12A* | rs485497^A^ | Increased susceptibility [18]. |
|  | rs583911^G^ | Increased susceptibility [18]. |
| *BLK* | rs2736345^G^ | Increased susceptibility [18]. |
|  | rs2729935^A^ | Increased susceptibility [18]. |
|  | rs6998387^A^ | Increased susceptibility [18]. |
| *CXCR5* | rs7119038^G^ | Decreased susceptibility [18]. |
|  | rs4936443^C^ | Decreased susceptibility [18]. |
| *TNIP1* | rs6579837^T^ | Increased susceptibility [18]. |
|  | rs7732451^G^ | Increased susceptibility [18]. |
| *IL10* | rs1800896^G^-rs1800871^C^-rs1800872^C^  (*IL10* −1082, −819, −592 haplotype) | Increased susceptibility [19, 20]. |
|  | rs1800896^A^-rs1800871^C^-rs1800872^C^  (*IL10* −1082, −819, −592 haplotype) | Decreased susceptibility [19]. |
|  | rs1800896^A^-rs1800871^T^-rs1800872^A^  (*IL10* −1082, −819, −592 haplotype) | Increased susceptibility [19]. |
| *IL4RA* | ARSPRV haplotype | Higher frequency of parotid gland enlargement, trend towards higher prevalence of positive immunological parameters [21]. |
| *MBL2* | MBL-low genotypes | Lower prevalence of extraglandular involvement, autoantibody positivity [22]. |
| *TNF* | rs1800629^A^ (−308) | Increased risk [20, 23]. |
| *SFTPD* | rs721917^G^ | Higher prevalence of renal involvement [24]. |

**References**

1. Cenit MC, Martínez-Florensa M, Consuegra M, et al (2014) Analysis of ancestral and functionally relevant CD5 variants in systemic lupus erythematosus patients. PLoS One 9:e113090. https://doi.org/10.1371/journal.pone.0113090

2. Carnero-Montoro E, Bonet L, Engelken J, et al (2012) Evolutionary and functional evidence for positive selection at the human CD5 immune receptor gene. Mol Biol Evol 29:811–823. https://doi.org/10.1093/molbev/msr251

3. Swaminathan B, Cuapio A, Alloza I, et al (2013) Fine Mapping and Functional Analysis of the Multiple Sclerosis Risk Gene CD6. PLoS One 8:e62376. https://doi.org/10.1371/journal.pone.0062376

4. Eyre S, Bowes J, Diogo D, et al (2012) High-density genetic mapping identifies new susceptibility loci for rheumatoid arthritis. Nat Genet 44:1336–1340. https://doi.org/10.1038/ng.2462

5. Castro MAA, Oliveira MI, Nunes RJ, et al (2007) Extracellular Isoforms of CD6 Generated by Alternative Splicing Regulate Targeting of CD6 to the Immunological Synapse. J Immunol 178:4351–4361. https://doi.org/10.4049/jimmunol.178.7.4351

6. Kofler DM, Severson CA, Mousissian N, et al (2011) The CD6 Multiple Sclerosis Susceptibility Allele Is Associated with Alterations in CD4+ T Cell Proliferation. J Immunol 187:3286–3291. https://doi.org/10.4049/jimmunol.1100626

7. De Jager PL, Jia X, Wang J, et al (2009) Meta-analysis of genome scans and replication identify CD6, IRF8 and TNFRSF1A as new multiple sclerosis susceptibility loci. Nat Genet 41:776–782. https://doi.org/10.1038/ng.401

8. Swaminathan B, Matesanz F, Cavanillas ML, et al (2010) Validation of the CD6 and TNFRSF1A loci as risk factors for multiple sclerosis in Spain. J Neuroimmunol 223:100–103. https://doi.org/10.1016/j.jneuroim.2010.03.020

9. Leppä V, Surakka I, Tienari PJ, et al (2011) The genetic association of variants in CD6, TNFRSF1A and IRF8 to multiple sclerosis: A multicenter case-control study. PLoS One 6:e18813. https://doi.org/10.1371/journal.pone.0018813

10. Consuegra-Fernández M, Julià M, Martínez-Florensa M, et al (2018) Genetic and experimental evidence for the involvement of the CD6 lymphocyte receptor in psoriasis. Cell Mol Immunol 15:898–906. https://doi.org/10.1038/cmi.2017.119

11. Johnson BA, Wang J, Taylor EM, et al (2010) Multiple sclerosis susceptibility alleles in African Americans. Genes Immun 11:343–350. https://doi.org/10.1038/gene.2009.81

12. Zheng M, Zhang L, Yu H, et al (2016) Genetic polymorphisms of cell adhesion molecules in Behçet’s disease in a Chinese Han population. Sci Rep 6:24974. https://doi.org/10.1038/srep24974

13. Jostins L, Ripke S, Weersma RK, et al (2012) Host–microbe interactions have shaped the genetic architecture of inflammatory bowel disease. Nature 491:119–124. https://doi.org/10.1038/nature11582

14. Ellinghaus D, Jostins L, Spain SL, et al (2016) Analysis of five chronic inflammatory diseases identifies 27 new associations and highlights disease-specific patterns at shared loci. Nat Genet 48:510–518. https://doi.org/10.1038/ng.3528

15. Wagner M, Bilinska M, Pokryszko-Dragan A, et al (2014) ALCAM and CD6 - multiple sclerosis risk factors. J Neuroimmunol 276:98–103. https://doi.org/10.1016/j.jneuroim.2014.08.621

16. Zhou P, Du LF, Lv GQ, et al (2011) Functional polymorphisms in CD166/ALCAM gene associated with increased risk for breast cancer in a Chinese population. Breast Cancer Res Treat 128:527–534. https://doi.org/10.1007/s10549-011-1365-x

17. Cruz-Tapias P, Rojas-Villarraga A, Maier-Moore S, Anaya JM (2012) HLA and Sjögren’s syndrome susceptibility. A meta-analysis of worldwide studies. Autoimmun Rev 11:281–287. https://doi.org/10.1016/j.autrev.2011.10.002

18. Lessard CJ, Li H, Adrianto I, et al (2013) Variants at multiple loci implicated in both innate and adaptive immune responses are associated with Sjögren’s syndrome. Nat Genet 45:1284–1294. https://doi.org/10.1038/ng.2792

19. Hulkkonen J, Pertovaara M, Antonen J, et al (2001) Genetic association between interleukin-10 promoter region polymorphisms and primary Sjögren’s syndrome. Arthritis Rheum 44:176–179. https://doi.org/10.1002/1529-0131(200101)44:1<176::AID-ANR23>3.0.CO;2-K

20. Qin B, Wang J, Liang Y, et al (2013) The Association between TNF-α, IL-10 Gene Polymorphisms and Primary Sjögren’s Syndrome: A Meta-Analysis and Systemic Review. PLoS One 8:e63401. https://doi.org/10.1371/journal.pone.0063401

21. Ramos-Casals M, Font J, Brito-Zeron P, et al (2004) Interleukin-4 receptor alpha polymorphisms in primary Sjögren’s syndrome. Clin Exp Rheumatol 22:374

22. Ramos-Casals M, Brito-Zerón P, Soria N, et al (2009) Mannose-binding lectin-low genotypes are associated with milder systemic and immunological disease expression in primary Sjögren’s syndrome. Rheumatology 48:65–69. https://doi.org/10.1093/rheumatology/ken411

23. Ben-Eli H, Gomel N, Aframian DJ, et al (2019) SNP variations in IL10, TNFα and TNFAIP3 genes in patients with dry eye syndrome and Sjogren’s syndrome. J Inflamm (United Kingdom) 16:1–6. https://doi.org/10.1186/s12950-019-0209-z

24. Soto-Cárdenas MJ, Gandía M, Brito-Zerón P, et al (2015) Etiopathogenic role of surfactant protein D in the clinical and immunological expression of primary sjögren syndrome. J Rheumatol 42:111–118. https://doi.org/10.3899/jrheum.140394
